# Pertussis toxin neutralizing antibody response after an acellular booster vaccination in Dutch and Finnish participants of different age groups

**DOI:** 10.1080/22221751.2022.2053364

**Published:** 2022-03-30

**Authors:** Aapo Knuutila, Pauline Versteegen, Alex-Mikael Barkoff, Pieter van Gageldonk, Jussi Mertsola, Guy Berbers, Qiushui He, PERISCOPE Consortium

**Affiliations:** a Institute of Biomedicine, University of Turku, Turku, Finland; b Centre for Infectious Disease Control, National Institute for Public Health and the Environment, Bilthoven, The Netherlands; c Department of Paediatrics and Adolescent Medicine, Turku University Hospital, Turku, Finland; d InFLAMES Research Flagship Center, University of Turku, Turku, Finland

**Keywords:** Adolescent, adult, child, neutralizing antibody, pertussis, pertussis toxin, vaccination

## Abstract

Pertussis incidence has increased in many countries and the disease occurs among all age groups, suggesting the need for booster immunizations through life. In addition to determining the concentration of anti-pertussis toxin (PT) antibodies, the ability of PT neutralizing antibodies (PTNAs) could be used to assess vaccine responses.

Altogether 258 participants [7–10-year-old (N = 73), 11–15-year-old (N = 85), 20–35-year-old (N = 50) and 60–70-year-old (N = 50)] were included. Sera were collected before, one month, and one year after a single dose of a three pertussis component containing acellular pertussis vaccine. The adolescents were primed in childhood either by acellular or whole-cell vaccination. PTNA titres were determined by a Chinese hamster ovary cell assay and anti-PT IgG/IgA antibody concentrations by multiplex immunoassay.

In all age groups, a significant increase in levels of PTNAs and anti-PT IgG was observed one month after vaccination and remained at least two-fold higher one year post-booster, in comparison to pre-booster. Young adults had the lowest response. The strongest increase in PTNAs was observed in participants who had ≥10 IU/mL concentration of anti-PT IgG antibodies pre-booster. At pre-booster, whole-cell-primed adolescents had higher PTNAs than acellular-primed peers (*p* = 0.047). One year post-booster, the Finnish whole-cell-primed adolescents had a higher level of PTNAs than acellular-primed adolescents (*p* = 0.049), however, this was not observed in Dutch adolescents. In conclusion, PTNAs increased after vaccination in all age groups, and the strongest increase was related to the presence of high pre-booster antibodies.

## Introduction

**Figure 1.**
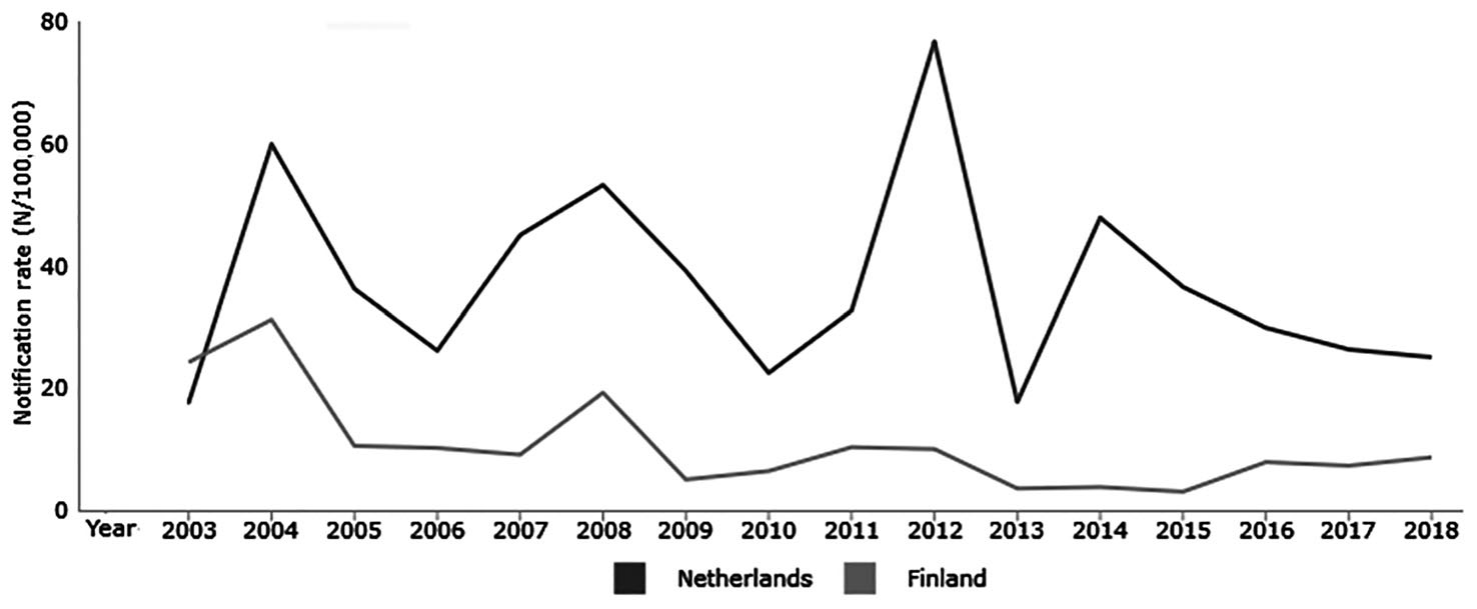
Laboratory confirmed pertussis notification rates in Finland and the Netherlands [5]. The study participants for this study were recruited from late 2017 to early 2019.

Pertussis is an acute respiratory infection in humans, caused by the Gram-negative bacterium *Bordetella pertussis*. Since the introduction of whole-cell pertussis vaccines (wPv) in the 1940s, widespread vaccination of young children has reduced the incidence of pertussis. However, due to the high reactogenicity of wPvs, acellular pertussis vaccines (aPv) were developed in the early 1980s. Current aPvs contain a combination of purified proteins from *B. pertussis*: (detoxified) pertussis toxin (PT), filamentous hemagglutinin, pertactin and fimbriae 2/3. In 2005, aPvs were nationally implemented in the Netherlands and Finland. Afterward, the aPvs’ composition, and their priming and boosting schedules have often changed in both countries (Supplementary Figure 1). However, despite high vaccination coverage (>95%), a rise in notifications of pertussis in the Netherlands and many other European countries has been seen, with regular epidemic peaks observed every two to four years [1–3]. In particular, the pertussis incidence in the last decennium has increased in adolescents and adults. In contrast, in Finland, there have been considerably fewer notifications in the last 15 years since the implementation of aPvs [4] (Figure 1). One explanation for this might be the use of aPv boosters in adolescents and young adults in Finland.

Waning immunity to wPvs and especially to aPvs following primary immunization is thought to contribute to the sustained transmission of *B. pertussis* in the population. Protection against the disease was shown to wane soon after a fifth booster vaccination at 4–6 years of age, however, children primed with wPvs seemed to be better protected than aPv primed children [2, 6–8]. Booster studies in aPv/wPv-primed school children in the Netherlands corroborate this finding [9], resulting in higher antibody levels in wPv-primed children one year after booster vaccination. Although antibody levels are usually higher after aPv than after wPv priming [10], their concentration decreases relatively fast during the first years after vaccination [10–12]. *B. pertussis* antigen-specific antibodies could be related to protection against pertussis [13–15], but the presence of antibodies alone does not always confer protection against infection [11, 12, 16]. In addition, in the baboon model, aPv protected against the disease, but could not prevent bacterial transmission. In contrast, vaccination with wPv or previous infection with *B. pertussis* conferred protection to infection in baboons [17].

Many aPv efficacy studies are mainly based on detecting anti-PT IgG antibody concentrations. The neutralization activity of antibodies has been studied as the quantity of neutralizing antibodies to PT (PTNA), which induces the inhibition of clustering of the Chinese hamster ovary (CHO) cells [18]. Essentially, aPvs have demonstrated high post-vaccination PTNAs [19, 20], whereas wPvs induce considerably lower levels of PTNAs [21, 22]. This could be attributed to the overall magnitude of the induced concentration of anti-PT IgG antibodies and to the amount of PT in the vaccines [19, 23, 24]. Generally, a four-fold increase at one month post-vaccination or post-infection was considered as a significant increase [25], and despite a rapid decrease post-vaccination, the induced PTNAs were still detectable after two to five years [20, 26].

The majority of CHO-cell-based studies have shown a clear correlation between the concentration of anti-PT IgG antibodies and PTNA titres [24], concluding that basic ELISA measurements generally demonstrate the neutralization capacity of antibodies [23, 27, 28]. Thereafter, as CHO cell-based assays are laborious, prone to subjective result analysis and less sensitive in comparison to ELISAs [29], the majority of studies in the last few decades have left CHO-cell assays out of laboratory practice. However, the role of PTNAs may remain essential in more thorough analysis models in large clinical studies with multiple serological and humoral immunological parameters aiming for the search of potential biomarkers of immune responses to *B. pertussis* and its waning immunity. This study aimed to characterize the PTNA response to an aPv booster in children, adolescents, young adults and older adults, with different priming vaccination backgrounds in two countries with different epidemiological backgrounds for pertussis incidence.

## Materials and methods

### Study approval

This “Booster against pertussis” (BERT) study trial was registered at the EU Clinical Trial database (EudraCT number 2016-003678-42) and was approved by the Medical Research Ethics Committees United (MEC-U, NL60807.100.17-R17.039) in the Netherlands and by the MREC UTU (ETMK Dnro: 129/1800/2017) in Finland [30]. Written informed consent was obtained from all adult and adolescent participants and parents or legal guardians of children at the start of the study.

### Study design

Participants from the BERT study cohort [30] (N = 258) (Table 1) were included in 2017–2019 in Finland and the Netherlands, and received a booster dose of a Tdap3-IPV vaccine (Boostrix™-IPV - GlaxoSmithKline (GSK), Wavre, Belgium). Samples for this study were analyzed from pre-booster, one month after and one year after booster vaccination. All sera in this study were stored at −20 °C, and their anti-PT IgG and IgA antibodies were measured previously with a fluorescent-bead-based multiplex immunoassay at the National Institute for Public Health and the Environment, The Netherlands [30, 31].

**Table 1.** Study cohorts.

	Country	Age (Mean yr)	No. of study participants	No. of Female/Male	Primary vaccination*	Booster vaccinations*, age
Children	FI	9.0	37	18/19	aPv	4 years, aPv
	NL	8.5	36	18/18		
Adolescents (aP)	FI	12.5	19	7/12	aPv	4 years, aPv
	NL	12.4	25	17/8		
Adolescents (wP)	FI	15.0	18	11/7	wPv	2 and 6 years, aPv**
	NL	14.8	23	14/9		4 years, aPv
Young adults	FI	30.2	25	21/4	wPv	n/A
	NL	28.6	25	10/15		
Older adults	FI	64.2	25	21/4	wPv	n/A
	NL	65.9	25	14/11		

* Detailed vaccine compositions and schedules in Supplementary Figure 1.

** One participant was boosted by wPv at 2 years of age

### CHO cell assay

Titres of PTNAs were determined by a CHO cell assay at the University of Turku, Finland, and at the Capital Medical University, Beijing, China, as previously described [32, 33]. In brief: 5,000 or 10,000 CHO cells were mixed with 0.84 ng/mL of native PT (GlaxoSmithKline, Rixensart, Belgium), and with a two-step dilution series of serum (1:8–1:4096). The wells were evaluated visually after 24 h either by microscopy or by Incucyte or by IncucyteZoom (Essen Bioscience, Michigan, USA) instruments. The neutralizing titre was reported as the serum dilution in the last well without clusters. Each 96-well plate included three controls: 1) only PT and cells; 2) only testing sera and cells and 3) only cells.

### Statistics

The PTNA results were analyzed as neutralization titres and as the proportion of neutralization divided by anti-PT IgG concentration (IU/mL) [34]. Samples with titres below the assay cut-off “1:8” were arbitrarily attributed to “1:4” for analyses. For this study, a proportion value of <0.5 was chosen for a low neutralization proportion and >2.0 for a high proportion. Cases of >2.0 PTNAs per anti-PT IgG may be slightly overrepresented in pre-booster samples due to very low anti-PT IgG concentrations (less than 1 IU/mL) extrapolating these values. Data were analyzed using IBM SPSS statistics 25.0 software for Windows (IBM Corp., Armonk, NY, USA). The differences in distributions between the groups were tested with Mann–Whitney *U*-tests with Bonferroni corrections, and two-sided *p*-values less than 0.05 were considered statistically significant. The correlations of PTNA responses to the overall anti-PT IgG and IgA concentrations were calculated with the Pearson correlation coefficient. Wilcoxon signed rank test was used for the comparison of median titres between the pre- and post-booster time points.

## Results

PTNA titres increased in all age groups during one month and remained higher than baseline after one year (Wilcoxon *p* < 0.05) (Table 2). Participants with a higher anti-PT IgG concentration pre-booster, regardless of study group or country, had a significantly higher anti-PT IgG and PTNA response after one month and after one year of vaccination (*p* < 0.05) (Table 3). The lowest pre-booster anti-PT IgG concentration to demonstrate this effect was 10 IU/mL, which was determined comparing adjacent ordinal variable values over a range of two-fold IU/mL intervals, starting from 5 IU /ml. This effect was not observed with PTNA per IgG proportions. In 120 out of 123 (97.6%) Finnish and 119 out of 134 (88.8%) Dutch participants at least two-fold higher PTNAs were observed one month post-booster in comparison to pre-booster. The PTNA titres remained at least two-fold higher in 85.4% of Finnish and 50.0% of Dutch participants one year post-booster. In comparison, sole anti-PT IgG concentrations increased two-fold at one month post-booster in 95.9% of Finnish and 96.3% Dutch participants. One year post-booster 72.4% of Finnish and 67.2% of Dutch participants had at least two-fold higher anti-PT IgG concentrations compared to pre-booster, respectively. Of note, the participants without a two-fold increase in PTNAs or anti-PT IgGs were present in all age groups, and the majority of these participants had very high (>50 IU) anti-PT IgG or high PTNA titres (≥32) pre-booster.

**Table 2.** Geometric mean values of anti-PT IgG concentrations (IU/mL), PTNA titres, and PTNA per anti-PT IgG ratios before the booster and one month (1M) and one year (1Y) post-booster.

Cohort	Country	PTNAs			anti-PT IgG			PTNAs per anti-PT IgG		
		Pre	1M	1Y	Pre	1M	1Y	Pre	1M	1Y
All	FI	14*	136*	40	11	159	42	1.28*	0.85*	0.95
	NL	22*	171*	37	10	139	35	2.12*	1.24*	1.19
Children	FI	18	198	47*	16	199	39	1.13	1.00*	1.22*
	NL	20	210	22*	12	147	30	1.72	1.43*	0.96*
Adolescents (aP)	FI	12*	154	36	10	187	43	1.11	0.82	0.82
	NL	28*	203	51	17	140	41	1.61	1.47	1.29
Adolescents (wP)	FI	19*	181*	69	16	219	68	1.16*	0.83*	1.02
	NL	43*	272*	49	11	161	48	3.81*	1.69*	1.19
Young adults	FI	8	68	20	5	99	27	1.49*	0.68	0.78
	NL	11	68	23	3	99	22	3.39*	0.68	1.33
Older adults	FI	16	118	43	10	132	51	1.58	0.89	0.86
	NL	21	179	56	15	156	43	1.36	1.15	1.31

*Significant difference between the countries, Mann-Whitney U-test, *p* < 0.05

**Table 3.** Comparison of geometrical mean values of anti-PT IgG concentrations (IU/mL) and PTNA titres before and after booster vaccination in study participants with lower or higher pre-booster anti-PT IgG concentrations. Statistical significant differences (Mann-Whitney U-test, *p* < 0.05) between the pre-booster anti-PT IgG classifications were observed at all time points and both variables in all age groups.

	Country	Pre-booster anti-PT IgG	Number of participants	anti-			PTNA		
				pre	PT IgG 1M	1Y	pre	1M	1Y
Children	FI	≥10 IU/mL	27	27	231	49	25	208	49
		<10 IU/mL	10	4	128	20	7	169	30
	NL	≥10 IU/mL	18	32	211	56	42	287	44
		<10 IU/mL	18	4	109	19	16	105	7
Adolescents	FI	≥10 IU/mL	23	27	230	91	24	201	74
		<10 IU/mL	14	4	163	23	7	122	25
	NL	≥10 IU/mL	32	31	181	59	63	299	67
		<10 IU/mL	16	3	100	20	9	163	22
Young adults	FI	≥10 IU/mL	7	33	185	67	43	141	64
		<10 IU/mL	18	3	78	19	4	51	13
	NL	≥10 IU/mL	4	38	487	105	91	362	152
		<10 IU/mL	21	2	73	17	7	49	16
Older adults	FI	≥10 IU/mL	13	33	292	109	32	243	79
		<10 IU/mL	12	3	56	22	8	54	23
	NL	≥10 IU/mL	18	26	227	60	27	228	57*
		<10 IU/mL	7	4	59	17	11	95	29*

*The only pair without statistically significant difference, Mann-Whitney U-test, *p* = 0.169

**Figure 2.**
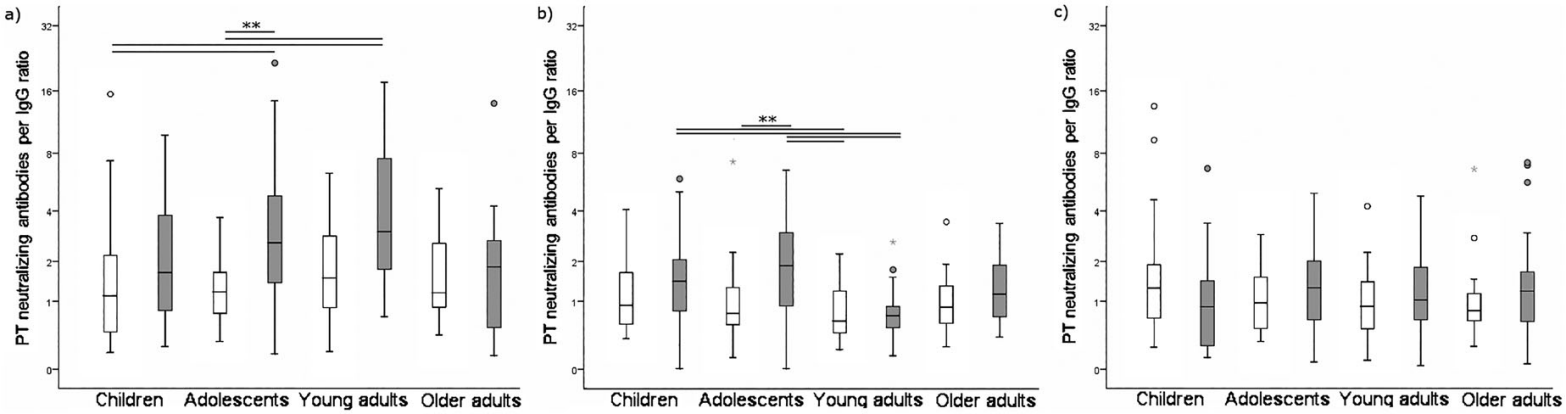
PTNA per anti-PT IgG ratio distribution at a) pre-booster, b) one month c) one year after vaccination. Light boxplots = Finland, Grey = Netherlands. The box plots demonstrate the median, quartile range, and 1.5 times the quartile range of PTNA per anti-PT IgG ratios. Mann-Whitney *U*-test **p < 0.01.

In terms of PTNA per anti-PT IgG ratio, the ratios decreased significantly in both countries from pre-booster to one month and one year post-booster (Wilcoxon *p* < 0.05), and the ratios remained at the same level from one month to one year after vaccination (Wilcoxon *p* = 0.84) (Figure 2, Table 2). Both PTNAs, as well as PTNA per anti-PT IgG proportions pre- and one month post-booster, were significantly higher in the Netherlands compared to Finland (*p* < 0.006). The number of individuals possessing overall low PTNA per anti-PT IgG proportions at post-booster increased from pre-booster (Table 4) in both countries, however, the magnitude of change was higher in the Dutch participants. On the contrary, the number of individuals possessing overall high PTNA per anti-PT IgG proportions at post-booster decreased from pre-booster. After one month post-booster, 29.3% of Finnish and 32.1% of Dutch participants had an improved PTNA per anti-PT IgG ratio in comparison to pre-booster. The ratio remained higher after one year in 33.9% of Finnish and 26.1% of Dutch participants. Particularly, Dutch children and adolescents had declining PTNA per anti-PT IgG titres after vaccination, whereas Finnish children and adolescents had an increasing or constant level of neutralization between one month and one year post- booster (Table 2).

**Table 4.** Comparison of PTNA per anti-PT IgG proportions in participants from Finland and the Netherlands.

PTNA per anti-PT IgG	Country	Number of participants (%)		
		Pre	One month	One year
<0.5	FI	19 (15.4%)	23 (18.5%)	24 (19.7%)
	NL	8 (6.0%)	12 (9.1%)	28 (21.1%)
0.5–2.0	FI	69 (56.1%)	84 (67.7%)	82 (67.2%)
	NL	53 (39.8%)	80 (60.6%)	79 (59.4%)
>2.0	FI	35 (28.5%)	17 (13.7%)	16 (13.1%)
	NL	72 (53.7%)	40 (30.3%)	26 (19.5%)

**Figure 3.**
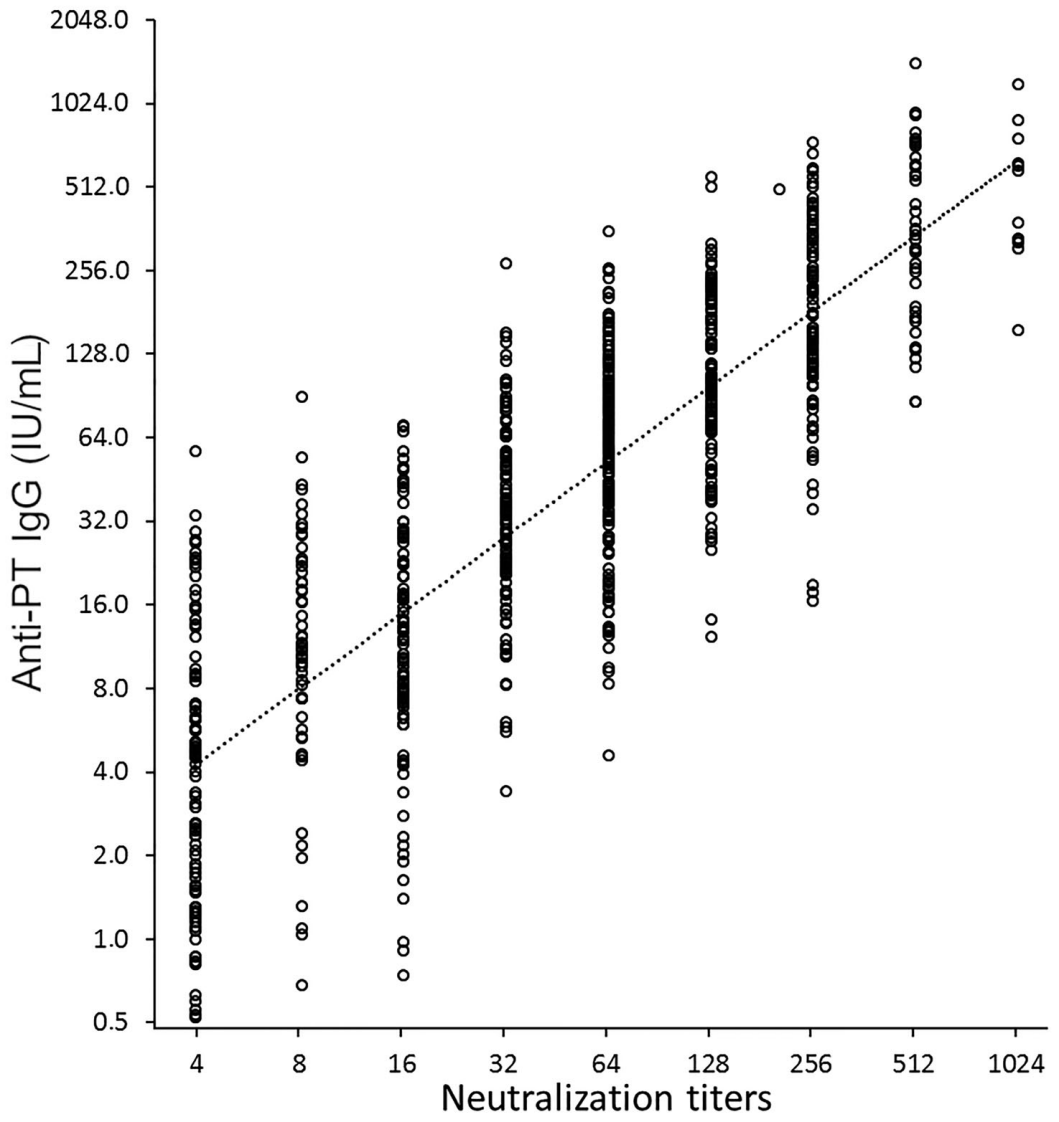
The correlation between overall anti-PT IgG concentration and PT neutralizing antibody titres was high (Pearson R = 0.829). All study samples at all three time points are presented (N = 769).

The PTNA titres were found to correlate significantly with overall antibody concentrations of anti-PT IgG antibodies (1:1 ratio, Pearson R = 0.829) (Figure 3). However, there was no correlation between age and PTNA titres. In the older adults, a high correlation between anti-PT IgA antibodies and PTNAs at one month was noted (R = 0.755), as well as at one year post-booster (R = 0.576), implying a possible role of anti-PT IgA in neutralization. Correlations in the other age cohorts were all below 0.5 at all time points. Consequently, it could be interpreted that, particularly for older adults, the calculated PTNA per anti-PT Ig ratios would slightly decrease with the implementation of IgA to overall IgG concentrations (Table 2).

The adolescents with wPv background had significantly higher pre-booster PTNA per anti-PT IgG ratios in comparison to aPv background adolescents (*p* = 0.047) when data from both countries were combined. One year after the booster, Finnish wPv-primed adolescents had significantly higher PTNAs than aPv-primed adolescents (*p* = 0.049), however, this was not observed in Dutch adolescents. Of note, the Dutch adolescents had significantly higher PTNAs pre-booster and one month after vaccination in comparison to Finnish adolescents (*p* < 0.01) (Table 2). Consequently, when data were analyzed individually per country, no consistent differences were noticed between the vaccination backgrounds at any time points with either PTNAs or PTNA per anti-PT IgG proportions.

## Discussion

Vaccination-induced antibodies play an important role in the protection against infection with *B. pertussis*. Antibodies can provide protection in many distinct ways. In this study, the functionality of vaccination-induced antibodies was assessed through the ability to neutralize PT in different age groups. All study groups in the Netherlands had higher PTNA titres and PTNA per anti-PT IgG ratios in pre-booster samples in comparison to Finland. Based on the reported incidence (Figure 1) and recent serosurveillance data [2, 3, 35, 36], pertussis is indeed more prevalent in the Netherlands than in Finland. Additionally, the pertussis primary vaccination coverage in 2017 was 94% in the Netherlands and 99% in Finland, and the children receive different amounts of vaccinations (Supplementary Figure 1). However, the epidemiological differences of pertussis between both countries were not reflected in the level of anti-PT IgG concentrations in pre-booster samples. Although in general, not all pertussis patients or infected individuals develop high PTNAs or anti-PT IgG concentrations [25, 33], high pre-booster PTNAs, particularly noticed in the Dutch adolescents, may be an indication of natural infection.

Long-lasting memory from previous exposure to *B. pertussis* or vaccination may present a challenge for the evaluation of vaccine responses. Although PTNAs increased significantly in all age groups after vaccination, the induced PTNA titres were significantly higher in participants with a pre-booster concentration of at least 10 IU/mL anti-PT IgG antibodies. A similar phenomenon was reported earlier with an aPv booster in Finnish adolescents [37]. Interestingly, if only subjects who had less than 10 anti-PT IgG IU/ml pre-booster (Table 3) were compared, the anti-PT IgG responses were close to equal in all age groups in both countries one year post vaccination. After one month, the children and adolescents had a more vigorous response in comparison to the adults. Conversely, if subjects had more than 10 anti-PT IgG IU/ml pre-booster, the differences between anti-PT IgGs could be over two fold after one year. Thereafter it could be deduced that the individuals with high pre-booster anti-PT IgG greatly impact upon the variety and magnitude in the observed vaccination responses between the age cohorts. Only 11/50 participants in the young adult group had more than 10 IU/mL anti-PT IgG, suggesting less frequent exposure or completely waned immunological memory to PT (Supplementary Figure 1). This may shed some insight into why the young adult cohort responded poorly to the vaccine in comparison to the other age groups in this study [30]. A similar effect of pre-booster antibodies was observed with the same study participants when anti-PT memory and plasma B-cells were determined by ELISPOT assays (Versteegen, Barkoff, Pinto, unpublished work). Also, post-booster mucosal antibody response was found significantly higher in participants with evidence of prior infection (van Schuppen, unpublished work) in the Dutch aP primed adolescents.

This phenomenon was not observed in the proportions of PTNAs per anti-PT IgG one month or one year post-booster. In addition, the model of PTNA per anti-PT IgG implied that the proportion of vaccine-induced PTNAs remains the same from one month to one year after vaccination, which indicates that merely the quantity of PTNAs changes over time. Based on a recent study by Zhang et al. [33], the ratio of PTNAs to anti-PT IgG was slightly above a ratio of 1.0 in young pertussis patients with recent infection, and who had received at least three vaccine doses. Although the number of participants with a relatively low proportion of PTNAs per total anti-PT IgG was increased after boosting, the post-booster proportions (Table 2) were equally high as post-infection [33]. In this light, acellular booster vaccination produced sufficiently high proportions of PTNAs in all age groups. The high proportion of PTNAs per anti-PT IgG in pre-booster antibodies could be possibly related to the importance of maintaining these antibodies in blood circulation. It is uncertain whether a low PTNA per anti-PT IgG proportion is reflected on the quality or function of antibodies through epitope-specificity and affinity of the induced antibodies [24], or whether sera with low PTNAs per anti-PT IgG contain in return a lot of other antibody classes or antibodies which excel in other protective functions, such as opsonization. Thus far, anti-PT IgA PTNAs have mostly been speculated to play a minor role in neutralization overall in comparison to anti-PT IgG since aPvs induce hardly any anti-PT IgA response in comparison to anti-PT IgG [6, 25, 27, 30, 38]. In this study, a high correlation between anti-PT IgA to PTNA titres was noted in older adults, suggesting that IgA antibodies possess the function to neutralize PT.

Although differences were observed between different priming vaccination backgrounds in the adolescent cohort pre-booster, it was not possible to further demonstrate the effect of age or priming background on booster response consistently in both study countries. Of note, the wPv group in Finland had received five doses in childhood, whereas the aPv group had received only four (Supplementary Figure 1). However, in previous studies, no differences were noticed in PTNAs between children who received four or three doses of vaccines in childhood, either at one or at five years of age [26, 39]. Also, in the Dutch cohort, the pre-school aPv booster in the wPv group was administered two years earlier compared to their aPv background peers, whereas in Finland the time difference from the latest vaccination was close to six months. These factors may influence the outcome of aPv/wPv comparisons. Although the research setting for the evaluation of vaccination history was simple, the vaccination background in the adolescent age group appeared to be quite divergent, thereby making the results hard to interpret. Further, standardization and comparison of the PTNA results with earlier studies are challenging, since standards are rarely used in CHO-cell assays, and they have been found to vary up to four-fold between laboratories [40]. Despite the significant correlation between antibody concentrations and CHO-cell assays, strongly diverging results between anti-PT IgG concentration and PTNAs occur. Thereafter, a result obtained by one method could not be used to predict a concentration for the other method with accuracy in an individual serum sample [23, 27, 28, 33]. This is often remarked as an inconvenience for assay comparisons, but on the other hand, may as well highlight an alternative landmark for evaluating a successful vaccination response, which may have a significant impact at an individual level.

It can be concluded that the epidemiological differences between the two countries and inter-individual differences in pre-existing memory to pertussis have a significant influence on anti-PT IgG and PTNAs after vaccination. Our finding would suggest that a pre-booster anti-PT IgG concentration as low as 10 IU/mL works as an excellent predictor of successful vaccine response in terms of a high quantity as well as a good quality of anti-PT IgG response. Conversely, it could be considered that individuals with less than 10 IU/mL PT antibodies would have required either more frequent boosting, or that a single booster dose for those individuals was not enough to reactivate immunological memory. Pre-existing antibodies from an earlier infection or previous vaccination may very well be influenced by priming background and thereafter to the likelihood of being infected by *B. pertussis*. This was reflected particularly in the young adult study cohort of both countries, in which the participants had very low pre-booster anti-PT antibodies and in return, responded, at least in terms of quantity of PTNAs, weaker towards the vaccination. However, the relative proportion of PTNAs among overall post-booster antibodies between the different study groups did not differ significantly. On the other hand, based on PTNA titres, adolescents in the Netherlands were likely to be more frequently exposed to *B. pertussis*. In addition to PTNAs, other functional antibodies to PT e.g. antibodies responsible for bacteria killing and opsonophagocytosis as well as antibody avidity and B-cell memory should be considered. Altogether, our results stress the importance to determine PT neutralizing antibodies for the assessment of functional antibodies after aPv vaccinations. Additionally, there is a clear indication for future studies to present and analyze data more explicitly in regards subjects’ pre-antibody levels in order to visualize the effect of subject background on reported vaccination responses.

## Supplementary Material

Supplemental Material

## Data sharing statement

Individual participant data that underlie the results reported in this article, have been de-identified and deposited in the central database of the PERISCOPE Consortium and can be accessed by a request to the PERISCOPE management team.
