# Supplementary material for: Pertussis toxin neutralizing antibody response after an acellular booster vaccination in Dutch and Finnish participants of different age groups
Source: Emerg Microbes Infect. 2022 Mar 30;11(1):956–63. doi: 10.1080/22221751.2022.2053364 (PMC8973383; doi:10.1080/22221751.2022.2053364)
Supplement: Supplemental Material [file TEMI_A_2053364_SM5881.docx]

Supplementary Figure 1. Pertussis vaccine schedules and vaccines in Finland and the Netherlands. Modified from Versteegen et al [30].

**
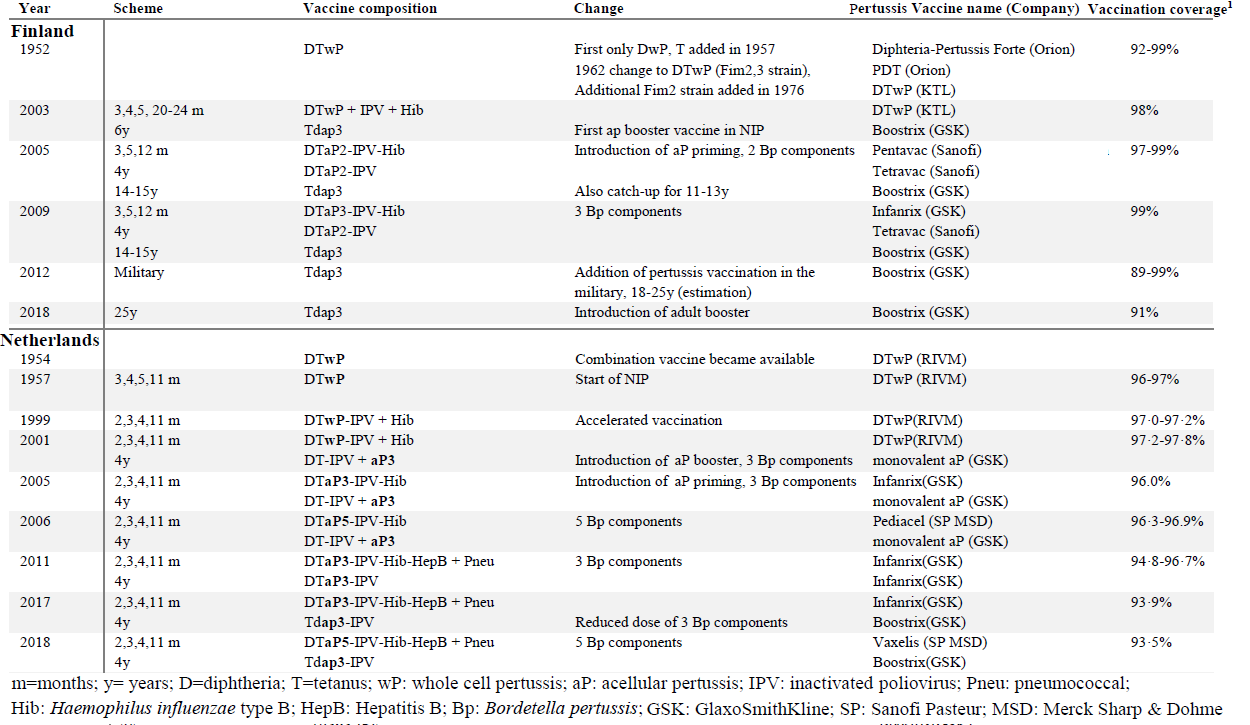
**
